# Timing of tummy-time introduction and developmental outcomes during the first year of life: an exploratory pilot study

**DOI:** 10.3389/fped.2026.1923077

**Published:** 2026-10-19

**Authors:** Anne H. Zachry, Faria Abdullah, Rana Assfoura, Taylor Berke, Markous Boushra, Amira Ghaly, Matthew Khalil, Lauren Woods, Chenhao Zhao

**Affiliations:** 1 Department of Occupational Therapy, The University of Tennessee Health Sciences (UTHS), Memphis, TN, United States; 2 Division of General Pediatrics, The University of Tennessee Health Sciences (UTHS), Memphis, TN, United States; 3 Division of General Pediatrics, Le Bonheur Children’s Hospital, Memphis, TN, United States; 4 Department of Obstetrics and Gynecology, The University of Tennessee Health Sciences (UTHS), Knoxville, TN, United States; 5 Department of Pediatrics, The University of Tennessee Health Sciences (UTHS), Memphis, TN, United States; 6 Department of Anesthesiology, The University of Tennessee Health Sciences (UTHS), Knoxville, TN, United States; 7 Department of General Surgery, University of Louisville School of Medicine, Louisville, KY, United States; 8 Department of Preventive Medicine, The University of Tennessee Health Sciences (UTHS), Memphis, TN, United States

**Keywords:** caregivers, child development, infant, motor skills, prone position

## Abstract

Prior research has associated greater tummy-time exposure with infant development but less is known about whether the timing of initial tummy-time introduction is related to developmental outcomes. This exploratory longitudinal pilot study examined developmental patterns among 58 infants introduced to tummy time within the first 2 postnatal weeks, after the first 2 postnatal weeks, or not at all. Caregivers reported tummy-time introduction and monthly positioning and behavioral experiences. Development was assessed using the Ages and Stages Questionnaire, Third Edition (ASQ-3) at 3, 6, 9, and 12 months. Complete 5-domain ASQ-3 data were available for 32 infants at 3, 6, and 9 months and 30 infants at 12 months. Kruskal–Wallis tests compared the 3 exposure groups across 5 developmental domains and 4 assessment ages. Rank-based eta-squared effect sizes with 95% bootstrap confidence intervals (CIs) were reported, with Benjamini-Hochberg correction across 20 primary comparisons. Three comparisons had unadjusted *p*-values .05, but none remained statistically significant after correction. The largest observed effect was for communication at 9 months (*η*^2^_H_=0.275; 95% CI, 0.100–0.507), although CIs were generally wide. Substantial attrition, small and unequal groups, caregiver-reported exposure and outcomes, and potential residual confounding limited the ability to make inferences. These findings do not provide clear evidence of reproducible developmental differences according to the timing of tummy-time introduction. Larger prospective studies should evaluate timing alongside cumulative tummy-time exposure and broader developmental influences.

## 1 Introduction

Placing an infant in the prone position for play while awake and supervised, known as tummy time, has a number of benefits, such as preventing cranial asymmetry and promoting head control and motor skill development by strengthening an infant's neck, trunk, and upper extremities (1–6). Tummy time also allows an infant to explore and interact socially, which has the potential to promote the advancement of social and cognitive skills (7).

The American Academy of Pediatrics (AAP) recommends that infants be exposed to the prone position for play with supervision during the awake hours (3). Current AAP guidance recommends supervised tummy time while infants are awake, beginning early in infancy, with short sessions that are gradually increased to at least 15–30 min total per day by approximately 7 weeks of age (3).

A number of studies have examined the developmental benefits of prone positioning while awake during infancy. For example, a study conducted by Dudek-Shriber and Zelazny (1) examined the impact of tummy time on developmental milestone quality and achievement in a convenience sample of 100 caregivers of infants. When the infants were 4 months old, caregivers completed a questionnaire and reported the frequency and total amount of time their child spent in different positions over a 24-hour period. Motor development was assessed using the Alberta Infant Motor Scale. Results revealed that infants exposed to more tummy time achieved certain prone, supine, and sitting developmental milestones earlier compared to those with less tummy time. Similarly, a descriptive pilot study compared a cross-sectional sample of 66 infants to examine how sleep and play positioning related to gross motor development at 2, 4, and 6 months of age. At 2 months, infants who slept on their backs or sides and those who experienced 15 min or less of tummy time a day achieved certain gross motor milestones at lower rates than expected, while no significant differences were found at 4 or 6 months, suggesting that positioning may influence early gross motor development (4). Zhang et al. (6) followed infants during their first 6 months of life and found that tummy time frequency, duration, and infant preference for tummy time increased with age. Greater amounts of tummy time were associated with improved developmental outcomes, particularly at 4 and 6 months, suggesting that the amount of tummy time associated with optimal development may increase as infants get older.

Tummy time has also been examined in relation to cranial asymmetry and broader developmental outcomes. Zachry et al. (5) conducted a cross-sectional study in which parents of infants diagnosed with cranial asymmetry and parents of infants who were developing typically completed a survey that gathered data related to demographics, development, time spent in various developmental positions, and time spent in baby gear. The authors found that children diagnosed with cranial asymmetry were exposed to significantly less tummy time than children without the diagnosis. A systematic review by Hewitt et al. (2) included 16 studies with 4,237 infants from 8 countries examining the relationship between tummy time and infant health outcomes. Prone play time while awake was associated with greater gross motor and overall development, lower BMI z-scores, improved movement skills, and reduced brachycephaly. However, the evidence for several other developmental outcomes was mixed or showed no association. The authors noted that most studies were observational and had methodological limitations.

Beyond motor development, tummy time may provide infants with opportunities for exploration and social interaction. Buchanan et al. (7), using longitudinal data from a randomized controlled trial, examined associations between tummy-time practices and later movement behaviors, including active play, outdoor play, screen time, and sleep at 12 and 24 months of age. When the infants were 6 months of age, caregivers reported the age their infant first began practicing tummy time, as well as how many days per week the infant was exposed to tummy time. Initiation of tummy time was divided into <4 weeks and ≥4 weeks. The results revealed that initiating tummy time within the first 4 weeks of age on a daily basis was related to more movement behaviors and increased sleep duration in children at 12 and 24-months of age (7). Another longitudinal study using compositional data analysis investigated the relationship between sleep time, restrained time, supine time, and prone play time with development in 93 infants at the ages of 2, 4, and 6 months. Movement data were collected using a time-use diary. Movement behaviors were significantly associated with improved gross-motor, problem-solving, and overall development of infants at 6 months. Increased sleep and tummy time were related to increased development and earlier milestone achievement, while more time in the supine position was linked to poorer outcomes, with the results related to restrained time being mixed (8).

Across 3 studies, tummy time was associated with improved infant motor and developmental outcomes, although the studies had small sample sizes. Two intervention studies of infants aged 3–6 months found significant improvements in gross motor development following 2–3 weeks of tummy time exercises, with infants receiving tummy time demonstrating more advanced motor skills than those who did not or compared with their pre-intervention performance (9, 10). Similarly, a cross-sectional study of infants aged 6–12 months found that tummy time performed more than 1–2 times per week for 6–20 min per session was associated with better developmental outcomes, although earlier initiation and the number of daily sessions were not significantly associated with development (11).

Previously cited research generally supports an association between tummy time and infant development; however, these studies focused primarily on the amount, frequency, duration, and patterns of tummy time exposure, rather than the timing of its initial introduction. The timing of tummy time introduction may be developmentally relevant because prone positioning provides early opportunities for infants to practice antigravity head and trunk control, upper-extremity weight bearing, postural control, and active exploration. Introducing tummy time during the early postnatal period may provide additional opportunities to practice these foundational skills during a period of rapid development and may promote familiarity and tolerance of the prone position, potentially facilitating continued participation. Therefore, in addition to the amount of tummy time infants receive, the timing of initial exposure may be relevant to future developmental outcomes. However, whether earlier introduction is associated with measurable developmental advantages remains unclear.

It remains unclear whether introducing tummy time during the first weeks after birth is associated with different developmental outcomes compared with beginning later in infancy, which represents a gap in knowledge because clinical recommendations encourage supervised tummy time beginning early in infancy, yet there is limited evidence establishing whether earlier initiation itself confers a developmental advantage, independent of later or no tummy time participation. Examining developmental outcomes among infants who began tummy time within the first 2 weeks of life, after 2 weeks of age, or not at all provides an opportunity to address this understudied aspect of tummy time exposure. Understanding whether the timing of tummy time initiation is associated with later development may help strengthen the evidence base underlying clinical recommendations and provide more specific guidance to healthcare professionals and caregivers regarding when tummy time should be introduced.

### 1.1 Aim

The aim of this exploratory pilot study was to examine whether the timing of tummy time introduction, early (within the first 2 postnatal weeks) or later (after the first 2 postnatal weeks) introduction, or no introduction at all (never) was associated with differences in communication, gross motor, fine motor, problem-solving, and personal-social developmental outcomes during the first year of life. The primary objectives included the following: (1) characterize preliminary patterns in developmental outcomes across tummy time introduction groups; (2) estimate the magnitude and uncertainty of between-group differences; and (3) generate findings to inform the design and hypotheses of future adequately powered studies rather than to conduct confirmatory hypothesis testing.

## 2 Materials and methods

### 2.1 Study design and ethics

This longitudinal observational pilot study examined the relationship between the timing of tummy time introduction and infant developmental outcomes. The study protocol was approved by the Institutional Review Board of our university (Protocol #23-09209-XP), and informed consent was obtained from parents or caregivers before participation.

### 2.2 Participants and procedures

Participants were recruited through an informational social media post directing parents to a secure Qualtrics survey and through in-person recruitment at a pediatric practice. Screening procedures were conducted either in person or via Zoom or FaceTime or by an investigator in a private examination room. A total of 58 infants were initially enrolled. At baseline, parents completed a study-specific, non-validated caregiver questionnaire developed by the research team. The questionnaire collected infant date of birth and race; maternal and paternal age and education; caregiver marital status; household income; parity; infant sleep location; multiple-birth status; and prior caregiver education regarding infant positioning and screen exposure.

Caregivers reported the age at which tummy time was introduced using 5 prespecified response categories: 0–2 weeks; 2–4 weeks; 4–8 weeks; 2 months or older; or never. For analysis, these categories were collapsed into 3 groups: introduction within 0–2 weeks; introduction after 2 weeks; and never. The 0–2-week category represented introduction during the earliest postnatal period and most closely approximated recommendations to initiate tummy time soon after birth. Because relatively few infants were represented within the individual later-introduction categories, these categories were combined to support more meaningful group comparisons. Thus, the 2-week threshold represents an operational analytic definition rather than an established clinical or developmental cutoff.

Eligibility criteria were prespecified. Inclusion criteria of the participating parent or legal guardian included: (1) the ability to speak and read English; (2) age older than 18 years; (3) ownership of a cell phone with internet access and Zoom or FaceTime capability; and (4) usable working email address. Eligible infants included the following: 2 months of age or younger; was born at 37 or more weeks' gestation with a birthweight of at least 2,500 g; no parent-reported medical condition, health complication, or problem since birth that could affect movement behaviors or development; and no diagnosis of gastroesophageal reflux disease (GERD). Infants were excluded if they were regularly cared for by an adult other than their parent for 20 or more hours per week.

A link to the online ASQ-3 instrument was sent via text to caregivers when infants were 3, 6, 9, and 12 months of age, and the caregivers answered the ASQ-3 questions independently. This mode of administration was consistent across participants and assessment occasions.

### 2.3 Exposure and follow-up measures

The primary exposure was caregiver-reported age at first tummy-time introduction. The 58 enrolled infants were categorized into 3 groups: introduction within the first 2 postnatal weeks [Early tummy time (TT); *n* = 22]; introduction after the first 2 postnatal weeks, including 2–4 weeks, 4–8 weeks, or 2 months or older (Later TT; *n* = 21); and no reported tummy-time introduction (*N*ever TT; *n* = 15). The ‘never introduced’ classification indicates that the caregiver did not report intentionally introducing tummy time and should not be interpreted as indicating that the infant had no prone positioning or prone-play exposure. Monthly follow-up surveys were collected monthly at 2 to 7 months of age. Up to 2 reminder messages were sent when participants did not respond. Caregivers reported their infant's typical daily time spent in the following situations: during tummy time; while side-lying; seated in a car safety seat/carrier, swing, bouncy seat or exersaucer/jumper; during exposure to television, smartphone, and tablet/computer; and during interactive play. Each duration item used the following ordered categories: 1 = 0 min; 2 = 1–15 min; 3 = 16–30 min; 4 = 31–45 min; 5 = 46–60 min; 6 = 61–90 min; and 7 = >90 min. Because category widths were unequal and the highest category was open-ended, responses were retained as ordinal category codes and were not converted to estimated minutes or summed across activities.

### 2.4 Developmental outcome measure

Developmental outcomes of all infants were assessed using the Ages and Stages Questionnaire, Third Edition (ASQ-3). The ASQ-3 is a parent-reported, standardized developmental screening questionnaire for infants and children ranging in age from 1 month to 5 years, 11 months that assesses the domains of communication, fine motor, gross motor, problem-solving, and personal social skills (12). The test-retest reliability of the ASQ-3 is 93% and validity ranges from 82%–88%, with a specificity of 85% and a sensitivity of 86% (13). Each domain yields a score that can be summarized continuously, while age- and domain-specific referral cutoffs are used for screening interpretation. The assessment referred to in this study as the 3-month assessment used the ASQ-3 4-month questionnaire, which is valid for infants aged 3 months, 0 days through 4 months, 30 days. The corresponding valid age windows were 5 months, 0 days through 6 months, 30 days for the 6-month questionnaire; 9 months, 0 days through 9 months, 30 days for the 9-month questionnaire; and 12 months, 0 days through 12 months, 30 days for the 12-month questionnaire. To ensure administration of the appropriate age-specific questionnaire, caregivers entered the date of assessment and the infant's date of birth after accessing the ASQ-3 website, and the system automatically administered the questionnaire corresponding to the infant's age. All infants included at a given nominal assessment point completed the same age-specific ASQ-3 questionnaire, and all assessments were completed within the valid age window for that questionnaire. Mean (SD) infant age at assessment was 3.18 (0.23), 6.17 (0.18), 9.21 (0.24), and 12.37 (0.28) months at the 3-, 6-, 9-, and 12-month assessments, respectively.

Verified referral cutoffs for the 4-month questionnaire were the following: 34.60 (Communication); 38.41 (Gross Motor); 29.62 (Fine Motor); 34.98 (Problem Solving); and 33.16 (Personal-Social). Cutoffs for the 6-month assessment were 29.65, 22.25, 25.14, 27.72, and 25.34, respectively; cutoffs for the 9-month assessment were 13.97, 17.82, 31.32, 28.72, and 18.91, respectively; and cutoffs for the 12-month assessment were 15.64, 21.49, 34.50, 27.32, and 21.73, respectively. Continuous domain scores were used for the primary exploratory comparisons to preserve information about score distributions and magnitude; a secondary sensitivity analysis classified scores at or below the age- and domain-specific referral cutoff. Because the ASQ-3 is a screening instrument rather than diagnostic instrument, and because it is also caregiver completed, small between-group differences may not reflect clinically meaningful developmental differences.

### 2.5 Statistical analysis

Descriptive statistics were used to summarize the baseline characteristics and ASQ-3 domain scores. ASQ-3 scores were summarized by tummy time introduction group using means, standard deviations, medians, interquartile ranges, and sample sizes. Because distributional assumptions were not consistently met and group sizes were small, Kruskal–Wallis rank-sum tests compared score distributions across the 3 tummy time groups for each of 5 domains at 3, 6, 9, and 12 months. Rank-based eta-squared (*η*^2^_H_) was calculated as (H−k + 1)/(n−k), and percentile 95% CIs were estimated using 2,000 stratified bootstrap samples. Benjamini-Hochberg false-discovery-rate correction was applied once across the 20 primary domain-by-time comparisons. Unadjusted *p*-values and Benjamini-Hochberg-adjusted (BH-adjusted) *p*-values are reported separately. For the primary comparisons, statistical significance after multiplicity correction was evaluated using BH-adjusted *p* < .05; findings with unadjusted *p* < .05 but BH-adjusted *p* ≥ .05 were considered nominal and exploratory. The 95% bootstrap CIs describe effect-size precision and were not adjusted for multiplicity.

A secondary sensitivity analysis classified infants as at or below the age- and domain-specific ASQ-3 referral cutoff versus above the cutoff and used Fisher exact tests with Benjamini-Hochberg correction applied separately across the 20 cutoff comparisons. Twelve-month attrition was examined by comparing baseline characteristics of infants with complete 5-domain 12-month outcomes versus without outcomes using absolute standardized mean differences (SMDs) and unadjusted exploratory *p*-values. Attrition comparisons were not included in the BH correction families; SMDs were emphasized when describing baseline imbalances. For monthly behavioral data, analyses were descriptive. Monthly records were broken down into 1 record per infant-month, and each infant was assigned a participant-level median ordinal category across available monthly surveys so that infants with more completed surveys did not receive greater weight. No missing monthly responses were imputed.

An *a priori* power calculation was not performed because the study was designed to be an exploratory pilot rather than a confirmatory hypothesis-testing study. A retrospective *post-hoc* power analysis was also not performed; observed effect sizes and CIs were used to characterize magnitude and precision.

## 3 Results

### 3.1 Participant flow and baseline sample

Tummy-time introduction data were available for all 58 enrolled infants: Early TT (within the first 2 postnatal weeks), *n* = 22; Later TT (after the first 2 postnatal weeks), *n* = 21; and Never TT (no reported introduction), *n* = 15. The same 32 infants contributed complete scores for all 5 ASQ-3 domains at each of the 3-, 6-, and 9-month assessments: Early TT, *n* = 15; Later TT, *n* = 9; and Never TT, *n* = 8. At 12 months, 30 of these infants had complete outcomes: Early TT, *n* = 14; Later TT, *n* = 8; and Never TT, *n* = 8 (Figure 1). These assessment-specific sample sizes apply to every domain comparison at the corresponding age (Table 1 and Figure 2). Baseline characteristics by tummy-time group are provided in Supplementary Table S1.

**Figure 1:**
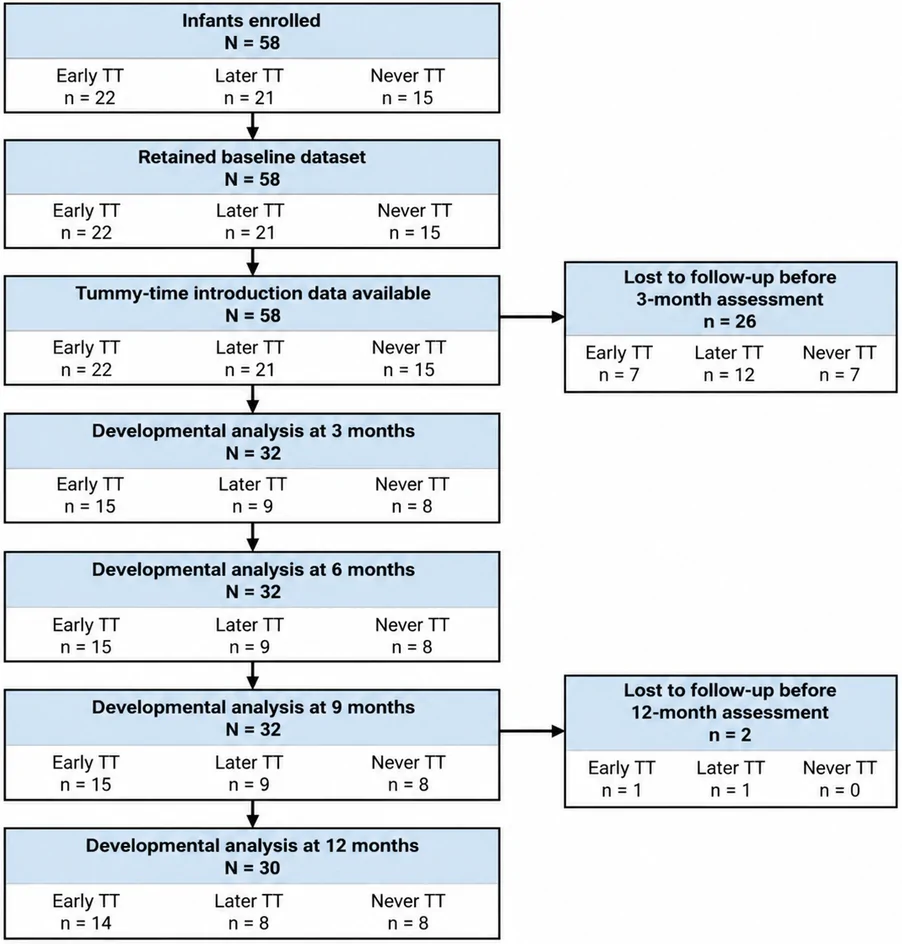
Flow diagram showing participant numbers across study time points. At enrollment, the Early TT, Later TT, and Never TT groups included 22, 21, and 15 infants, respectively (total *N* = 58). The same 32 infants contributed complete 5-domain ASQ-3 data at each of 3, 6, and 9-month intervals: Early TT, *n* = 15; Later TT, *n* = 9; and Never TT, *n* = 8. At 12 months, 30 of these infants contributed complete outcomes: Early TT, *n* = 14; Later TT, *n* = 8; and Never TT, *n* = 8. Early TT denotes introduction within the first 2 postnatal weeks; Later TT denotes introduction after the first 2 postnatal weeks; Never TT denotes no reported introduction. Parents of infants classified as lost to follow-up did not respond to telephone calls, text messages, or emails.

**Table 1:** Primary ASQ-3 comparisons across tummy-time introduction groups.

Month	Domain	Group n E/L/N (Total)	Kruskal–Wallis H statistic	Degrees of freedom	Unadjusted *p*-value	BH-adjusted *p*-value	Eta-squared H statistic (95% CI)
3	Communication	15/9/8 (32)	4.58	2	0.101	0.484	0.089 (0.000–0.431)
3	Gross motor	15/9/8 (32)	0.98	2	0.612	0.815	0.000 (0.000–0.290)
3	Fine motor	15/9/8 (32)	1.15	2	0.564	0.815	0.000 (0.000–0.295)
3	Problem solving	15/9/8 (32)	0.20	2	0.903	0.903	0.000 (0.000–0.254)
3	Personal-social	15/9/8 (32)	0.80	2	0.669	0.836	0.000 (0.000–0.241)
6	Communication	15/9/8 (32)	6.50	2	0.039	0.258	0.155 (0.004–0.458)
6	Gross motor	15/9/8 (32)	1.47	2	0.478	0.815	0.000 (0.000–0.336)
6	Fine motor	15/9/8 (32)	1.06	2	0.590	0.815	0.000 (0.000–0.266)
6	Problem solving	15/9/8 (32)	0.49	2	0.781	0.903	0.000 (0.000–0.217)
6	Personal-social	15/9/8 (32)	1.58	2	0.453	0.815	0.000 (0.000–0.329)
9	Communication	15/9/8 (32)	9.98	2	0.007	0.136	0.275 (0.100–0.507)
9	Gross motor	15/9/8 (32)	4.22	2	0.121	0.484	0.077 (0.000–0.393)
9	Fine motor	15/9/8 (32)	2.42	2	0.298	0.815	0.015 (0.000–0.312)
9	Problem solving	15/9/8 (32)	1.85	2	0.397	0.815	0.000 (0.000–0.324)
9	Personal-social	15/9/8 (32)	8.17	2	0.017	0.168	0.213 (0.041–0.497)
12	Communication	14/8/8 (30)	1.17	2	0.556	0.815	0.000 (0.000–0.346)
12	Gross motor	14/8/8 (30)	3.28	2	0.194	0.647	0.047 (0.000–0.386)
12	Fine motor	14/8/8 (30)	1.87	2	0.392	0.815	0.000 (0.000–0.342)
12	Problem solving	14/8/8 (30)	0.28	2	0.868	0.903	0.000 (0.000–0.222)
12	Personal-social	14/8/8 (30)	0.31	2	0.858	0.903	0.000 (0.000–0.238)

E, Early TT (within the first 2 postnatal weeks); L, Later TT (after the first 2 postnatal weeks); N, Never TT (no reported introduction). Counts give Early/Later/Never group sizes, with the total in parentheses. The same 32 infants contributed all 5 domain scores at 3, 6, and 9 months; 30 contributed all 5 domain scores at 12 months. BH, Benjamini-Hochberg correction applied once across all 20 primary domain-by-age comparisons. Unadjusted *p*-values are exploratory. No comparison had BH-adjusted *p* < .05. The 95% bootstrap CIs were not adjusted for multiplicity.

**Figure 2:**
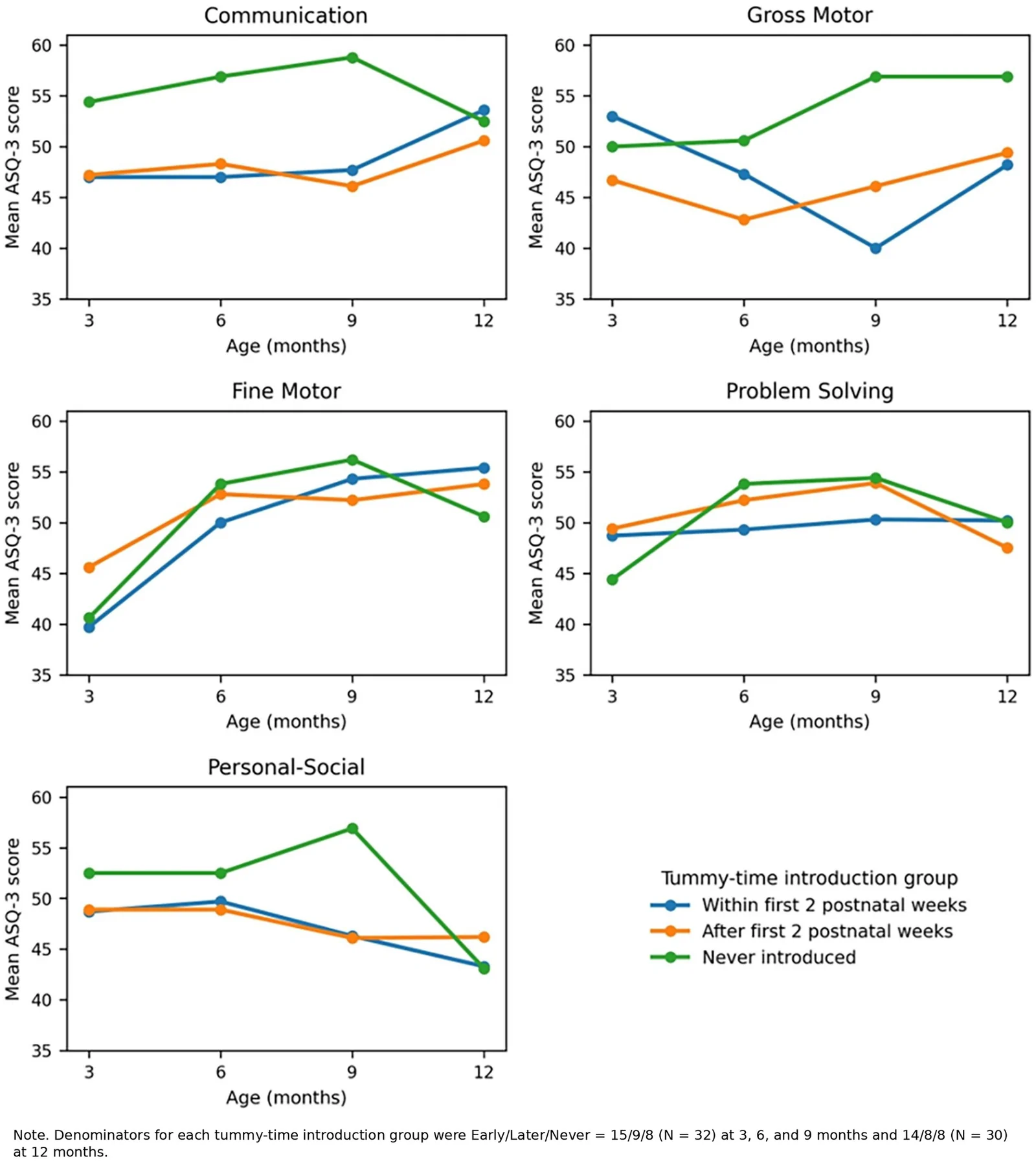
ASQ-3 domain scores at 3, 6, 9, and 12 months by timing of tummy-time introduction. Early TT denotes introduction within the first 2 postnatal weeks; Later TT denotes introduction after the first 2 postnatal weeks; Never TT denotes no reported introduction. Higher scores indicate more developmental skills reported within each domain. For every domain at each of 3, 6, and 9-month intervals, sample sizes were Early TT, *n* = 15; Later TT, *n* = 9; and Never TT, *n* = 8 (total *N* = 32). At 12 months, sample sizes were Early TT, *n* = 14; Later TT, *n* = 8; and Never TT, *n* = 8 (total *N* = 30). Nominal findings were observed for communication at 6 months (unadjusted *p* = .039; BH-adjusted *p* = .258), communication at 9 months (unadjusted *p* = .007; BH-adjusted *p* = .136), and personal-social skills at 9 months (unadjusted *p* = .017; BH-adjusted *p* = .168). BH denotes Benjamini-Hochberg correction across all 20 primary domain-by-age comparisons. None of the results were statistically significant after multiplicity correction; nominal findings were exploratory.

### 3.2 Primary developmental outcomes

Across 5 ASQ-3 domains and 4 ages, 20 omnibus Kruskal–Wallis comparisons were conducted. Three comparisons had nominal unadjusted *p*-values below.05: communication at 6 months (unadjusted *p* = .039; BH-adjusted *p* = .258), communication at 9 months (unadjusted *p* = .007; BH-adjusted *p* = .136), and personal-social skills at 9 months (unadjusted *p* = .017; BH-adjusted *p* = .168). None of the 20 primary comparisons was statistically significant after BH correction; these nominal findings are exploratory (Table 1 and Figure 2). The largest observed rank-based effect was for communication at 9 months (*η*^2^_H_ = 0.275; 95% CI, 0.100–0.507), followed by personal-social skills at 9 months (*η*^2^_H_ = 0.213; 95% CI, 0.041–0.497) and communication at 6 months (*η*^2^_H_ = 0.155; 95% CI, 0.004–0.458). CIs were wide, reflecting substantial uncertainty in this small sample. The remaining effect-size estimates were small or near zero, with CIs that generally included zero (Table 1).

The 9-month personal-social comparison was nominal and exploratory (unadjusted *p* = .017; BH-adjusted *p* = .168). The never-introduced group had the highest observed mean [56.9; standard deviation (SD), 4.6], compared with the within-first-2-weeks group (mean, 46.3; SD, 9.9) and after-2-weeks group (mean, 46.1; SD, 9.6). Because the omnibus Kruskal–Wallis test does not establish a specific pairwise contrast and the BH-adjusted result was not statistically significant, this finding is treated as exploratory rather than evidence of a meaningful early-versus-later effect.

During reanalysis for the revised manuscript, the 9-month analytic sample was updated from 27 to 32 infants after complete records not previously included in the original analysis were identified. Rerunning the Kruskal–Wallis test using the corrected analytic sample yielded a raw *p*-value of.017 rather than the previously reported value of.046. The addition of the Communication domain increased the number of comparisons included in the Benjamini-Hochberg correction from 16 to 20, but it did not itself affect the raw personal-social *p*-value.

### 3.3 Secondary ASQ-3 cutoff analysis

In the secondary cutoff-based sensitivity analysis, few infants scored at or below the age- and domain-specific ASQ-3 referral cutoffs, and no between-group Fisher exact test results remained statistically significant after separate Benjamini-Hochberg correction across the 20 cutoff comparisons. Detailed cutoff results are provided in Supplementary Table S2.

### 3.4 Attrition analysis

At 12 months, complete developmental outcome data were available for 30 of 58 enrolled infants (51.7%). Baseline comparisons between participants with and without 12-month outcome data showed potentially meaningful imbalances in infant age (SMD, 0.42), infant gender (SMD, 0.36), race (SMD, 0.49), birth order (SMD, 0.34), household income (SMD, 0.68), caregiver marital status (SMD, 0.68), maternal education (SMD, 0.93), paternal education (SMD, 0.57), and tummy-time introduction group (SMD, 0.42). Household size (SMD, 0.02) and multiple-birth status (SMD, 0.03) were similar. Unadjusted exploratory *p*-values were.022 for caregiver marital status and.004 for maternal education. These attrition comparisons were not corrected for multiple testing, so these *p*-values do not establish multiplicity-adjusted statistical significance; interpretation emphasizes the SMDs, sparse cells, and small sample (Table 2).

**Table 2:** Baseline characteristics by 12-month outcome availability.

Characteristic	12-month outcome available (*n* = 30)	12-month outcome not available (*n* = 28)	Absolute SMD	*Unadjusted* *p-value*
Infant age at baseline, weeks, mean (SD)	2.5 (1.8)	1.9 (1.0)	0.42	0.262
Household size, mean (SD)	3.9 (1.3)	3.9 (1.0)	0.02	0.874
Missing, *n* (%)	2 (6.7)	4 (14.3)	–	–
Infant gender, *n* (%)			0.36	0.289
Male	16 (53.3)	10 (35.7)	–	–
Female	14 (46.7)	17 (60.7)	–	–
Missing	0 (0.0)	1 (3.6)	–	–
Infant race, *n* (%)			0.49	0.115
White	9 (30.0)	3 (10.7)	–	–
Black	20 (66.7)	21 (75.0)	–	–
Indian/Asian	1 (3.3)	4 (14.3)	–	–
Birth order, *n* (%)			0.34	0.458
Oldest	7 (23.3)	3 (10.7)	–	–
Second child	16 (53.3)	16 (57.1)	–	–
Third or later child	7 (23.3)	9 (32.1)	–	–
Multiple birth, *n* (%)			0.03	>0.999
Yes	4 (13.3)	4 (14.3)	–	–
No	26 (86.7)	24 (85.7)	–	–
Total household annual income, *n* (%)			0.68	0.134
$20,000 or less	11 (36.7)	10 (35.7)	–	–
$20,001–$30,000	4 (13.3)	7 (25.0)	–	–
$30,001–$40,000	3 (10.0)	3 (10.7)	–	–
$40,001–$50,000	0 (0.0)	2 (7.1)	–	–
$50,001–$100,000	4 (13.3)	5 (17.9)	–	–
Above $100,000	8 (26.7)	1 (3.6)	–	–
Caregiver marital status, *n* (%)			0.68	0.022
Single, never married	12 (40.0)	20 (71.4)	–	–
Married	13 (43.3)	4 (14.3)	–	–
Living with partner	5 (16.7)	2 (7.1)	–	–
Separated	0 (0.0)	1 (3.6)	–	–
Other	0 (0.0)	1 (3.6)	–	–
Mother's education, *n* (%)			0.93	0.004
Some high school	0 (0.0)	4 (14.3)	–	–
GED	1 (3.3)	0 (0.0)	–	–
High school graduate	8 (26.7)	11 (39.3)	–	–
Some college	7 (23.3)	9 (32.1)	–	–
Bachelor's degree	5 (16.7)	4 (14.3)	–	–
Master's degree or higher	9 (30.0)	0 (0.0)	–	–
Father's education, *n* (%)			0.57	0.218
Some high school	2 (6.7)	2 (7.1)	–	–
GED	2 (6.7)	2 (7.1)	–	–
High school graduate	9 (30.0)	16 (57.1)	–	–
Some college	5 (16.7)	5 (17.9)	–	–
Bachelor's degree	6 (20.0)	3 (10.7)	–	–
Master's degree or higher	4 (13.3)	0 (0.0)	–	–
Missing	2 (6.7)	0 (0.0)	–	–
Tummy-time introduction group, *n* (%)			0.42	0.270
Within first 2 postnatal weeks	14 (46.7)	8 (28.6)	–	–
After first 2 postnatal weeks	8 (26.7)	13 (46.4)	–	–
Never introduced	8 (26.7)	7 (25.0)	–	–

SMD, standardized mean difference. The *p*-values in this attrition table are unadjusted and exploratory; no multiplicity correction was applied. They are separate from the BH-adjusted primary ASQ-3 and secondary cutoff comparisons. Baseline imbalances are interpreted principally using SMDs, with attention to sparse categories and the small sample.

### 3.5 Monthly positioning, equipment, screen exposure, and interaction

The 32-infant cohort contributed 176 unique infant-month observations; each infant contributed 4 to 6 monthly surveys (median, 6). Participant-level median ordinal categories were broadly overlapping across tummy-time groups for tummy time, side-lying, car seat/carrier use, screen exposure, and interactive play, although some differences were visible for swing and bouncy-seat categories (Supplementary Table S3). These summaries are descriptive and do not establish confounding, mediation, or causal effects.

## 4 Discussion

This exploratory pilot study examined whether the timing of the introduction of tummy time during infancy was associated with communication, gross motor, fine motor, problem-solving, and personal-social development during the first year of life. The findings do not provide clear evidence of reproducible between-group differences. Although 3 omnibus comparisons had raw *p*-values below.05, none remained statistically significant after correction for 20 primary comparisons. The largest effect-size estimate occurred for communication at 9 months, but the CI was wide. Accordingly, the findings are better interpreted in terms of preliminary patterns and uncertainty than as evidence for either the presence or absence of an association.

A difference between groups was observed for personal-social scores at 9 months; however, this finding did not remain statistically significant after correction for multiple comparisons and was not observed at 3, 6, or 12 months. The never-introduced group had the highest mean score, while scores were similar for infants introduced to tummy time within the first 2 weeks and those introduced later. Therefore, this finding does not suggest an advantage of earlier tummy-time introduction. One possible explanation for the difference at 9 months is the rapid developmental change that occurs during this period. As infants develop independent sitting and become more mobile, they have more opportunities to explore objects, interact with caregivers, and engage with their environment. Independent sitting changes opportunities for social interaction and object exploration, and emerging motor skills have been associated with development in other areas, including communication and language (14–16). Differences in these developmental experiences may have influenced personal-social scores regardless of when tummy time was introduced. By 12 months, additional motor, social, and environmental experiences may have reduced developmental differences between the groups. Monthly caregiver reports also showed that several movement and interaction experiences were generally similar across tummy-time groups, suggesting that infants who began tummy time at different ages may have had similar experiences later in infancy. Therefore, the isolated 9-month finding should be interpreted cautiously and considered exploratory rather than evidence that the timing of tummy-time introduction influenced personal-social development.

The lack of a consistent gross motor pattern also requires cautious interpretation. Previous research has associated tummy time with postural control, upper-extremity strength, head control, and early motor milestones (9–11). However, the present study addressed a different question: whether developmental outcomes differed according to the timing of initial tummy-time introduction. A difference of only several weeks between the early- and later-introduction groups may have been too small to produce persistent differences detectable with a broad developmental screening instrument. In addition, infants who began tummy time later may have accumulated similar amounts of tummy time afterward, potentially reducing differences between the introduction groups. Therefore, subsequent tummy-time experiences should be considered when interpreting associations between the timing of introduction and developmental outcomes.

The moderately high scores observed at several time points in the never-introduced group were counter to the study premise but should be interpreted cautiously. This subgroup was very small (*n* = 8 in the developmental analyses), making its estimates particularly sensitive to sampling variability and selection. Uncontrolled differences in family characteristics, infant development, caregiver practices, or other movement opportunities could also contribute. Because the study was designed to examine timing of tummy-time introduction rather than the independent developmental effects of tummy time itself, comparisons involving the never-introduced group should not be interpreted as evidence for or against the developmental benefits of tummy time.

The monthly behavioral data provide additional context for interpreting associations between the timing of tummy-time introduction and developmental outcomes. Prior research has examined tummy-time duration and other movement behaviors in relation to development (2, 6–8). In the present study, caregiver-reported positioning, restrictive-equipment use, screen exposure, and interactive play were available during follow-up and are described across groups. However, the sample was too small to reliably adjust for multiple factors. Also, because many of these behaviors were measured after tummy time was introduced, they may have been influenced by tummy-time practices rather than representing preexisting differences between groups. The descriptive data provide useful context but cannot determine whether these factors influenced the relationship between tummy-time timing and developmental outcomes.

Current recommendations supporting supervised prone play should not be reconsidered based on these pilot findings because this study was not designed to evaluate the developmental effects of tummy time itself. Rather, the findings suggest that the timing of initial tummy-time introduction should be considered within the broader context of infants’ subsequent movement opportunities and caregiver-infant experiences. Future studies examining timing of introduction should also prospectively measure subsequent tummy-time duration, frequency, quality, and context, along with relevant developmental and environmental factors.

### 4.1 Limitations and recommendations for future research

Attrition in this study was substantial. Only 30 of 58 enrolled infants (51.7%) had complete 5-domain outcomes at 12 months. Baseline SMDs indicated imbalances between infants with and without 12-month data, particularly for maternal education (SMD, 0.93), household income (SMD, 0.68), caregiver marital status (SMD, 0.68), paternal education (SMD, 0.57), infant race (SMD, 0.49), and tummy-time introduction group (SMD, 0.42). These differences suggest that attrition may not have been entirely random and create a risk of attrition bias. The retained sample therefore may not fully represent the original cohort, and differential loss to follow-up could have affected the magnitude or direction of observed associations.

This study also relied heavily on caregiver report. The timing of tummy time introduction was based on retrospective caregiver report and not prospectively documented at the time of first exposure, introducing the possibility of recall or exposure-classification bias. However, the initial survey was completed early in infancy (1–8 weeks of age, with most infants 1–4 weeks old), which likely limited the length of the recall period for most participants. Tummy-time introduction was reported retrospectively and is subject to recall error, while monthly positioning and behavioral exposures were reported in broad, unequal-width categories with an open-ended highest category. Developmental outcomes were measured using the caregiver-completed ASQ-3. Obtaining both exposure and outcome information from caregivers introduces potential common-method bias, and the ASQ-3—although standardized and useful for developmental screening—may have limited sensitivity for detecting minor between-group differences in developmental performance. The secondary cutoff analysis did not reveal adjusted between-group differences, but small counts limited inference.

The small and unequal group sizes produced imprecise estimates and wide CIs. Nonsignificant findings therefore should not be interpreted as demonstrating no association. In addition, the broad later-introduction category combined infants first exposed at different ages, and the temporal contrast between the early and some of the later participants may have been only a few weeks. Tummy-time practices following initial introduction were not standardized, and the study could not ensure comparable duration, quality, caregiver involvement, or consistency across groups. These factors could reduce observable differences attributable specifically to timing.

Infant development is multifactorial. Potential influences include temperament and tolerance for prone positioning, growth, birth order, parental education and experience, socioeconomic conditions, social support, caregiver-infant interaction, opportunities for unrestricted movement, restrictive-equipment use, screen exposure, sleep, and environmental stimulation. Some of these characteristics were measured, but the sample was too small to support a stable multivariable model with multiple covariates. Residual confounding is therefore likely. Future confirmatory studies should use larger and more diverse samples, prospectively defined exposure categories, objective or independently observed measures where feasible, detailed longitudinal measurement of tummy-time dose and quality, and prespecified covariate-adjusted analyses.

An *a priori* power calculation was also not appropriate for the purpose of this exploratory pilot study, and a retrospective *post-hoc* power analysis was not used because effect estimates and CIs characterize the observed magnitude and precision more directly. For future planning, investigators should select a clinically meaningful target effect before data collection. Assuming a future confirmatory/inferential study with 3 similar groups like our study, a double-sided *α* level of.05, with a power 80%, and a moderate effect size (Cohen's f = 0.25), the number of participants needed would be approximately 53 participants per group or 159 participants in total. This calculation is intended as an ideal planning benchmark. If considering possible 20% attrition, a future inferential study would need to recruit around 200 participants. Investigators planning future inferential studies could use effect-size estimates from this pilot study to finish their sample size calculation.

Strengths of this study include the longitudinal design, repeated assessment across 5 developmental domains, examination of an understudied aspect of tummy time, reporting of effect sizes with CIs and multiplicity-adjusted *p*-values, and explicit evaluation of attrition and monthly behavioral exposures.

## 5 Conclusion

In this exploratory pilot sample, we did not identify reproducible differences in ASQ-3 developmental outcomes according to whether tummy time was introduced within the first 2 postnatal weeks, after the first 2 postnatal weeks, or not at all. The findings are uncertain because of small and unequal groups, wide CIs, substantial and potentially selective attrition, caregiver-reported measures, and residual confounding. These findings should not be interpreted as evidence that the timing of tummy-time introduction has no relationship with development. The study also was not designed to determine the independent developmental effects of tummy time itself; rather, the results highlight the need to examine timing of introduction within the broader context of tummy-time practices, movement opportunities, caregiver interaction, and environmental influences. Larger prospective studies are needed to more precisely determine whether the timing of tummy-time introduction is associated with developmental outcomes.

## Data Availability

The raw data supporting the conclusions of this article will be made available by the authors, without undue reservation.
