# Supplementary material for: Timing of tummy-time introduction and developmental outcomes during the first year of life: an exploratory pilot study
Source: Front Pediatr. 2026 Oct 19;14:1923077. doi: 10.3389/fped.2026.1923077 (PMC13724422; doi:10.3389/fped.2026.1923077)
Supplement: Supplementary file 1 [file Table1.docx]

**Supplementary Table 1**. Baseline characteristics by tummy-time introduction group.

| **Characteristic** | **Overall**  **(N = 58)** | **Within first 2 postnatal weeks  (n = 22)** | **After first  2 postnatal weeks  (n = 21)** | **Never introduced  (n = 15)** |
| --- | --- | --- | --- | --- |
| **Infant age at baseline, weeks (X)** | 2.2 (1.5) | 2.5 (2.1) | 2.3 (1.1) | 1.6 (0.8) |
| **Household size, X (X)** | 3.9 (1.2) | 4.1 (1.2) | 3.9 (0.8) | 3.6 (1.5) |
| Missing, n (%) | 6 (10.3) | 3 (13.6) | 1 (4.8) | 2 (13.3) |
| Infant gender, n (%) |  |  |  |  |
| Male | 26 (44.8) | 9 (40.9) | 8 (38.1) | 9 (60.0) |
| Female | 31 (53.4) | 12 (54.5) | 13 (61.9) | 6 (40.0) |
| Missing | 1 (1.7) | 1 (4.5) | 0 (0.0) | 0 (0.0) |
| Infant race, n (%) |  |  |  |  |
| White | 12 (20.7) | 8 (36.4) | 4 (19.0) | 0 (0.0) |
| Black | 41 (70.7) | 13 (59.1) | 15 (71.4) | 13 (86.7) |
| Indian/Asian | 5 (8.6) | 1 (4.5) | 2 (9.5) | 2 (13.3) |
| Birth order, n (%) |  |  |  |  |
| Oldest | 10 (17.2) | 5 (22.7) | 2 (9.5) | 3 (20.0) |
| Second child | 32 (55.2) | 12 (54.5) | 14 (66.7) | 6 (40.0) |
| Third or later child | 16 (27.6) | 5 (22.7) | 5 (23.8) | 6 (40.0) |
| Multiple birth, n (%) |  |  |  |  |
| Yes | 8 (13.8) | 4 (18.2) | 4 (19.0) | 0 (0.0) |
| No | 50 (86.2) | 18 (81.8) | 17 (81.0) | 15 (100.0) |
| Total household annual income, n (%) |  |  |  |  |
| $20,000 or less | 21 (36.2) | 5 (22.7) | 7 (33.3) | 9 (60.0) |
| $20,001–$30,000 | 11 (19.0) | 4 (18.2) | 5 (23.8) | 2 (13.3) |
| $30,001–$40,000 | 6 (10.3) | 2 (9.1) | 2 (9.5) | 2 (13.3) |
| $40,001–$50,000 | 2 (3.4) | 0 (0.0) | 1 (4.8) | 1 (6.7) |
| $50,001–$100,000 | 9 (15.5) | 5 (22.7) | 4 (19.0) | 0 (0.0) |
| Above $100,000 | 9 (15.5) | 6 (27.3) | 2 (9.5) | 1 (6.7) |
| Caregiver marital status, n (%) |  |  |  |  |
| Single, never married | 32 (55.2) | 8 (36.4) | 14 (66.7) | 10 (66.7) |
| Married | 17 (29.3) | 10 (45.5) | 6 (28.6) | 1 (6.7) |
| Living with partner | 7 (12.1) | 4 (18.2) | 1 (4.8) | 2 (13.3) |
| Separated | 1 (1.7) | 0 (0.0) | 0 (0.0) | 1 (6.7) |
| Other | 1 (1.7) | 0 (0.0) | 0 (0.0) | 1 (6.7) |
| Mother's education, n (%) |  |  |  |  |
| Some high school | 4 (6.9) | 1 (4.5) | 1 (4.8) | 2 (13.3) |
| GED | 1 (1.7) | 0 (0.0) | 0 (0.0) | 1 (6.7) |
| High school graduate | 19 (32.8) | 3 (13.6) | 11 (52.4) | 5 (33.3) |
| Some college | 16 (27.6) | 9 (40.9) | 3 (14.3) | 4 (26.7) |
| Bachelor's degree | 9 (15.5) | 1 (4.5) | 5 (23.8) | 3 (20.0) |
| Master's degree or higher | 9 (15.5) | 8 (36.4) | 1 (4.8) | 0 (0.0) |
| Father's education, n (%) |  |  |  |  |
| Some high school | 4 (6.9) | 0 (0.0) | 2 (9.5) | 2 (13.3) |
| GED | 4 (6.9) | 2 (9.1) | 1 (4.8) | 1 (6.7) |
| High school graduate | 25 (43.1) | 6 (27.3) | 11 (52) | 8 (53.3) |
| Some college | 10 (17.2) | 5 (22.7) | 4 (19.0) | 1 (6.7) |
| Bachelor's degree | 9 (15.5) | 5 (22.7) | 3 (14.3) | 1 (6.7) |
| Master's degree or higher | 4 (6.9) | 4 (18.2) | 0 (0.0) | 0 (0.0) |
| Missing | 2 (3.4) | 0 (0.0) | 0 (0.0) | 2 (13.3) |

GED = General Educational Development certification.

**Supplementary Table 2.** ASQ-3 referral-cutoff sensitivity analysis.

| **Month** | **Domain** | **ASQ-3 referral cutoffs** | **Participants scoring at or below cutoffs per tummy-time introduction group,  No./group total (%)** | | | **Fisher  *p*-value** | **BH-adjusted *p*-value** |
| --- | --- | --- | --- | --- | --- | --- | --- |
|  |  |  | **Early** | **Later** | **Never** |  |  |
| 3 | Communication | 34.60 | 1/15 (6.7) | 1/9 (11.1) | 0/8 (0.0) | 1.000 | 1.000 |
| 3 | Gross motor | 38.41 | 0/15 (0.0) | 1/9 (11.1) | 2/8 (25.0) | 0.079 | 1.000 |
| 3 | Fine motor | 29.62 | 3/15 (20.0) | 1/9 (11.1) | 3/8 (37.5) | 0.481 | 1.000 |
| 3 | Problem solving | 34.98 | 1/15 (6.7) | 0/9 (0.0) | 2/8 (25.0) | 0.222 | 1.000 |
| 3 | Personal-social | 33.16 | 1/15 (6.7) | 1/9 (11.1) | 0/8 (0.0) | 1.000 | 1.000 |
| 6 | Communication | 29.65 | 0/15 (0.0) | 0/9 (0.0) | 0/8 (0.0) | 1.000 | 1.000 |
| 6 | Gross motor | 22.25 | 1/15 (6.7) | 2/9 (22.2) | 1/8 (12.5) | 0.790 | 1.000 |
| 6 | Fine motor | 25.14 | 0/15 (0.0) | 0/9 (0.0) | 0/8 (0.0) | 1.000 | 1.000 |
| 6 | Problem solving | 27.72 | 1/15 (6.7) | 0/9 (0.0) | 0/8 (0.0) | 1.000 | 1.000 |
| 6 | Personal-social | 25.34 | 0/15 (0.0) | 0/9 (0.0) | 1/8 (12.5) | 0.250 | 1.000 |
| 9 | Communication | 13.97 | 0/15 (0.0) | 0/9 (0.0) | 0/8 (0.0) | 1.000 | 1.000 |
| 9 | Gross motor | 17.82 | 0/15 (0.0) | 1/9 (11.1) | 0/8 (0.0) | 0.531 | 1.000 |
| 9 | Fine motor | 31.32 | 0/15 (0.0) | 1/9 (11.1) | 1/8 (12.5) | 0.274 | 1.000 |
| 9 | Problem solving | 28.72 | 1/15 (6.7) | 0/9 (0.0) | 1/8 (12.5) | 0.728 | 1.000 |
| 9 | Personal-social | 18.91 | 0/15 (0.0) | 0/9 (0.0) | 0/8 (0.0) | 1.000 | 1.000 |
| 12 | Communication | 15.64 | 0/14 (0.0) | 0/8 (0.0) | 0/8 (0.0) | 1.000 | 1.000 |
| 12 | Gross motor | 21.49 | 0/14 (0.0) | 1/8 (12.5) | 0/8 (0.0) | 0.533 | 1.000 |
| 12 | Fine motor | 34.50 | 0/14 (0.0) | 0/8 (0.0) | 0/8 (0.0) | 1.000 | 1.000 |
| 12 | Problem solving | 27.32 | 0/14 (0.0) | 0/8 (0.0) | 0/8 (0.0) | 1.000 | 1.000 |
| 12 | Personal-social | 21.73 | 1/14 (7.1) | 1/8 (12.5) | 2/8 (25.0) | 0.787 | 1.000 |

ASQ-3 = Ages and Stages Questionnaire, Third Edition ; BH = Benjamini-Hochberg.

**Supplementary Table 3**. Monthly survey summaries by tummy-time introduction group, X (X).

| **Monthly measure** | **Overall**  **(N = 32)** | **Within first 2 postnatal weeks**  **(n = 15)** | **After first 2 postnatal weeks**  **(n = 9)** | **Never introduced**  **(n = 8)** |
| --- | --- | --- | --- | --- |
| **Monthly surveys completed per infant** | 6.0 (5.0, 6.0) | 6.0 (5.0, 6.0) | 6.0 (5.0, 6.0) | 6.0 (5.0, 6.0) |
| **Tummy time, typical category** | 3.8 (3.0, 4.3) | 3.5 (3.0, 4.5) | 4.0 (2.0, 5.0) | 4.0 (3.5, 4.0) |
| **Side-lying, typical category** | 3.0 (2.0, 4.0) | 3.0 (2.0, 4.0) | 3.0 (2.0, 3.0) | 3.5 (3.0, 4.5) |
| **Car safety seat/carrier, typical category** | 3.3 (3.0, 4.0) | 3.0 (3.0, 4.0) | 3.5 (3.0, 4.0) | 3.3 (3.0, 4.3) |
| **Swing, typical category** | 2.0 (1.0, 3.0) | 1.0 (1.0, 2.5) | 1.0 (1.0, 3.0) | 2.8 (1.5, 3.5) |
| **Bouncy seat, typical category** | 2.0 (1.0, 3.0) | 2.0 (1.0, 3.0) | 1.5 (1.0, 2.0) | 2.8 (1.3, 3.3) |
| **Exersaucer/jumper, typical category** | 1.0 (1.0, 1.5) | 1.0 (1.0, 2.0) | 1.0 (1.0, 1.0) | 1.0 (1.0, 1.3) |
| **Television exposure, typical category** | 2.0 (1.3, 2.5) | 2.0 (1.0, 2.5) | 2.0 (2.0, 2.5) | 2.0 (1.5, 2.3) |
| **Smartphone exposure, typical category** | 1.0 (1.0, 1.0) | 1.0 (1.0, 1.0) | 1.0 (1.0, 1.0) | 1.0 (1.0, 1.0) |
| **Tablet/computer exposure, typical category** | 1.0 (1.0, 1.0) | 1.0 (1.0, 1.0) | 1.0 (1.0, 1.0) | 1.0 (1.0, 1.0) |
| **Interactive play, typical category** | 5.3 (4.5, 7.0) | 5.5 (5.0, 7.0) | 5.0 (3.0, 7.0) | 5.8 (4.3, 7.0) |

**Note.** Monthly survey summaries are based on the 32 infants included in the 3-, 6-, and 9-month analyses (Early, n = 15; Later, n = 9; Never, n = 8). The 12-month ASQ-3 sample included 30 infants and is reported separately in Table 1 and Supplementary Table 2.
